# A Compact Aperture-Slot Antipodal Vivaldi Antenna for GPR Systems

**DOI:** 10.3390/s26030810

**Published:** 2026-01-26

**Authors:** Feng Shen, Ninghe Yang, Chao Xia, Tong Wan, Jiaheng Kang

**Affiliations:** 1 School of Instrumentation Science and Engineering, Harbin Institute of Technology, Harbin 150001, China; sf407@126.com (F.S.);; 2 School of Mechatronics Engineering, Harbin Institute of Technology, Harbin 150001, China; 23s136365@stu.hit.edu.cn

**Keywords:** GPR antenna design, ground penetrating radar, non-destructive testing (NDT)

## Abstract

Compact antennas with ultra-wideband operation and stable radiation are essential for portable and airborne ground-penetrating radar (GPR), yet miniaturization in the sub 3 GHz region is strongly constrained by the wavelength-driven aperture requirement and often leads to impedance discontinuity and radiation instability. This paper presents a compact aperture-slot antipodal Vivaldi antenna (AS-AVA) designed under a radiation stability-driven co-design strategy, where the miniaturization features are organized along the energy propagation path from the feed to the flared aperture. The proposed structure combines (i) aperture-slot current-path engineering with controlled meandering to extend the low-frequency edge, (ii) four tilted rectangular slots near the aperture to restrain excessive edge currents and suppress sidelobes, and (iii) back-loaded parasitic patches for coupling-based impedance refinement to eliminate residual mismatch pockets. A fabricated prototype on FR-4 (thickness 1.93 mm) occupies 111.15×156.82 mm^2^ and achieves a measured $S_{11}$ below −10 dB from 0.63 to 2.03 GHz (fractional bandwidth 105.26%). The measured realized gain increases from 2.1 to 7.5 dBi across the operating band, with stable far-field radiation patterns; the group delay measured over 0.6–2.1 GHz remains within 4–8 ns, indicating good time-domain fidelity for stepped-frequency continuous-wave (SFCW) operation. Finally, the antenna pair is integrated into an SFCW-GPR testbed and validated in sandbox and outdoor experiments, where buried metallic targets and a subgrade void produce clear B-scan signatures after standard processing. These results confirm that the proposed AS-AVA provides a practical trade-off among miniaturization, broadband matching, and radiation robustness for compact sub 3 GHz GPR platforms.

## 1. Introduction

Ground-penetrating radar (GPR) is a non-destructive testing technology that utilizes high-frequency electromagnetic waves for subsurface detection [1,2,3,4] and is a key enabler of GPR-based remote sensing, demanding compact antennas with enhanced time-domain fidelity [5]. Its applications span geological exploration [6,7,8], engineering inspection [9], and transmission line detection [10,11]. However, miniaturization is crucial for deployment in confined spaces (e.g., pipelines, disaster zones), where conventional antennas often fail to reconcile broadband performance with compactness [12]. As the core component, antenna size directly governs system portability, necessitating innovative design [13]. Vivaldi antennas, leveraging their tapered slot structure, achieve stable directivity and high-resolution detection over ultra-wideband (UWB) ranges while maintaining relatively small lateral dimensions [14], making them ideal for compact GPR systems [15]. Sun, H.H. et al. designed a compact UWB dual-polarized antenna for full-polarimetric GPR applications [16]; Cheng, K.X. et al. proposed a compact dual-polarized Vivaldi antenna with high gain, utilizing an array configuration of two Vivaldi elements to optimize bandwidth and radiation performance [17]. However, Vivaldi antenna miniaturization faces physical limitations due to inherent structural constraints, hindering further size reduction to meet modern GPR requirements [18]. To address this, the antipodal Vivaldi antenna (AVA), optimized from Vivaldi antennas, has emerged as a key solution [19]. By refining radiation structures and impedance matching, AVA significantly reduces lateral dimensions while expanding low-frequency response, maintaining UWB operation, high gain, and stable directivity, thereby becoming a core technology for compact GPR systems.

Researchers have extensively explored AVA miniaturization. Deng, J.-Y. et al. enhanced the bandwidth of an antipodal Vivaldi antenna via a double-ridged SIW transition [20]; Dixit, A.S. et al. proposed a highly compact antipodal Vivaldi antenna array for 5G millimeter-wave applications by slotting antenna edges and constructing a 1 × 4 array [21]. Liu, L. et al. proposed a slit-slot transmission line and a co-located tripolarized slit-slot antenna array for 2.45 GHz MIMO, achieving lower cross-polarization and higher isolation than conventional slot arrays [22]. Compared to conventional AVA, these studies achieved miniaturization through slotting, feed transition redesign, and antenna integration. However, challenges remain, including excessive gain variation across bands, signal distortion from antenna coupling, structural complexity, and further optimization potential in size reduction.

Recent studies have shown that Vivaldi antennas, including antipodal Vivaldi variants (AVAs), can be made compact while retaining competitive radiation performance in different regimes, including GPR-oriented designs and high-frequency arrays. Typical miniaturization and bandwidth-enhancement techniques rely on structural perturbations (e.g., slotting, meandering, and parasitic loading) together with careful feed/transition design. However, when the operating band is pushed to the sub 3 GHz range that is preferred for penetration in practical GPR, the wavelength-driven aperture requirement imposes much tighter constraints, and the above measures often reach limitations that go beyond reflection coefficient optimization.

Specifically, slotting or aggressive current-path perturbation can lower the band edge and reduce electrical size, but it may also strengthen edge currents and excite higher-order effects, leading to gain ripple and pattern instability (e.g., elevated sidelobes and increased backward radiation), which becomes more pronounced in the upper band under strict size constraints. Likewise, coupling-based parasitic refinements can locally deepen $S_{11}$, yet they may still struggle to maintain a single continuous matched band together with stable forward radiation in the low-frequency region if the aperture field distribution is not co-designed with the radiation mechanism. For GPR, such radiation instability is particularly detrimental because it increases clutter and weakens the reliability of target signatures. Therefore, a key gap remains: a compact sub 3 GHz AVA-based GPR antenna that simultaneously achieves broadband impedance continuity, stable gain, and controlled sidelobes under stringent size constraints.

To position our contribution, Ant.6 is compared with representative compact Vivaldi/AVA-type antennas in [10,11,16,17] in terms of operating regime, size, continuous −10 dB bandwidth, and radiation stability. Ant.6 provides continuous matching from 0.63 to 2.03 GHz (FBW ≈ 105%) within 111.15×156.82 mm^2^ (about 0.23λ×0.33λ at 0.63 GHz), and the measured realized gain varies from 2.1 to 7.5 dBi (ΔG=5.4 dB) while maintaining stable forward radiation and controlled sidelobes. These comparisons indicate that the present design is not a simple combination of slotting/meandering/parasitic loading; instead, it follows a radiation stability-driven co-design strategy that jointly targets impedance continuity and robust radiation behavior in the wavelength-constrained sub 3 GHz GPR band.

To fill this gap, we propose a compact aperture-slot antipodal Vivaldi antenna (AS-AVA) based on a radiation stability-driven co-design strategy. The modifications are organized along the feed-to-aperture energy propagation path: aperture-slot current-path engineering with controlled meandering extends the low-frequency edge; tilted slots near the flared aperture suppress excessive edge currents and reshape the aperture field distribution for sidelobe reduction and gain stabilization; and back-loaded parasitic patches provide coupling-based impedance refinement to remove residual mismatch pockets and reinforce forward radiation. The fabricated prototype achieves $S_{11}<-10$ dB over 0.63–2.03 GHz in a compact footprint of 111.15×156.82 mm^2^, with a measured peak realized gain of 7.47 dBi and stable radiation characteristics. The stepwise evolution (Ant.1–Ant.6), together with simulations, anechoic-chamber measurements, and sandbox/outdoor GPR experiments, verifies the effectiveness of the proposed strategy for compact sub 3 GHz AVA-based GPR antennas.

## 2. Antenna Design and Analysis

### 2.1. Antenna Geometry

**Figure 1:**
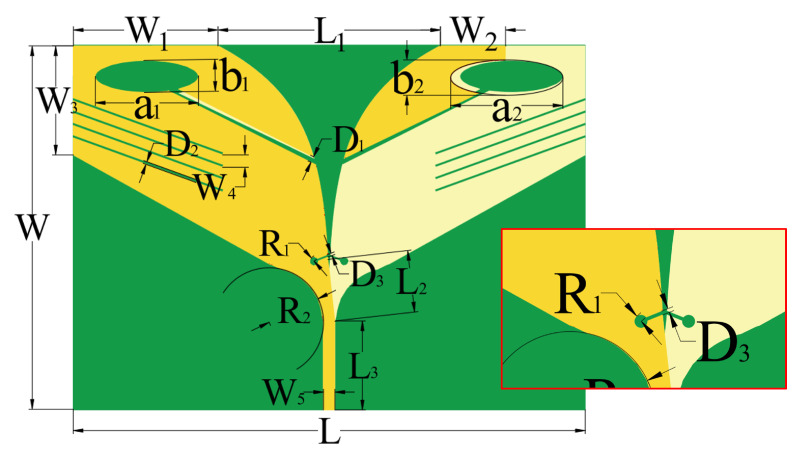
Geometry of the AS-AVA. (The geometrical dimensions are as follows: W = 111.15, W1 = 44.18, W2 = 19.94, W3 = 33.35, W4 = 3.78, W5 = 3.53, L = 156.82, L1 = 68.13, L2 = 18.92, L3 = 27.22, D1 = 1.87, D2 = 0.84, D3 = 0.77, R1 = 1.30, R2 = 16.67, a1 = 31.60, b1 = 9.78, a2 = 34.37, b2 = 10.80) (Unit: mm).

The geometric structure and dimensions of the AS-AVA are illustrated in Figure 1. It comprises a single dielectric substrate layer and dual metallic layers. The dielectric substrate is fabricated from FR-4 material with a thickness of 1.93 mm and a dielectric constant of 4.8. The tapered aperture of the antenna conforms to the profile defined by

(1) $$y=25.13\;\ln\;\left(0.04\;x+0.02\right)+103.01$$

**Figure 2:**
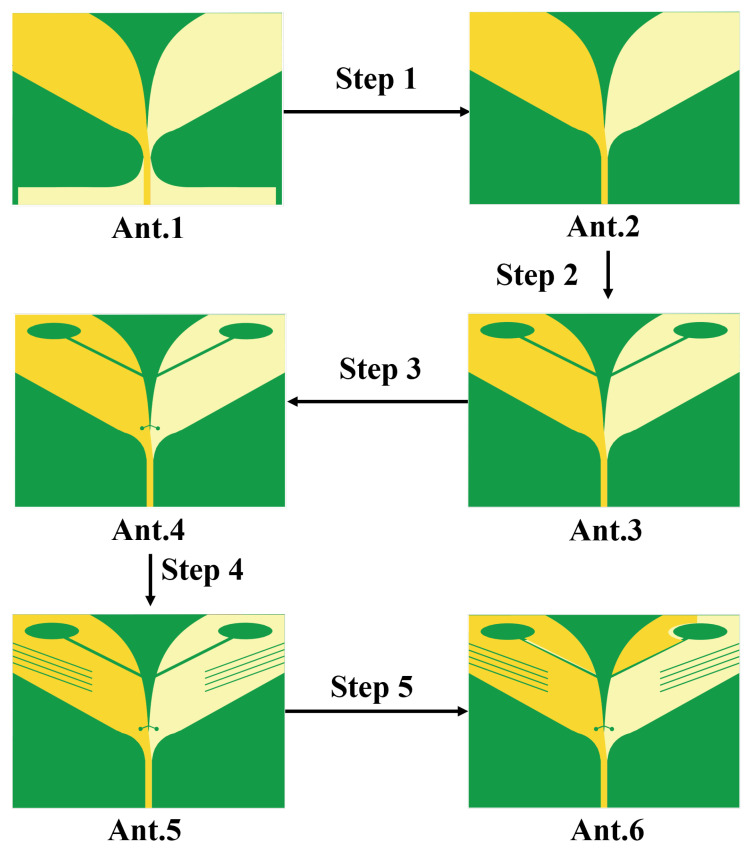
Progress of the AS-AVA antenna design.

The AS-AVA structure and dimensions are shown in Figure 1, where golden color represents the top copper layer, light yellow color represents the bottom copper layer, and green color represents the substrate, and the same in Figure 2. The AS-AVA achieves antenna performance optimization through apertures, slots, and parasitic structures. Specifically, one endpoint of the rectangular slot is positioned at the center of the elliptical structure, while the other endpoint aligns with the antenna’s central axis, thereby partitioning the right-side copper layer of the ellipse. A symmetric configuration is applied near the feedline on the inner side of the antenna. Additionally, four equally spaced, inclined rectangular slots are incorporated at the flared aperture of the AVA. Finally, a patch structure is symmetrically added to the opposite side of the antenna, forming a capacitive coupling with the copper layer on the adjacent side. This capacitive coupling mechanism effectively optimizes the antenna’s input impedance, ensuring robust impedance matching across the operational bandwidth.

### 2.2. Antenna Evolution

**Figure 3:**
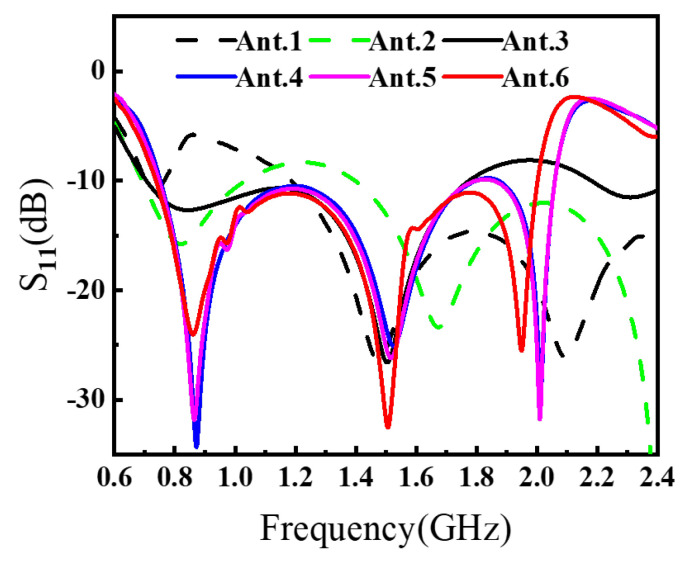
Simulated reflection coefficient $S_{11}$ of Ant.1–Ant.6 during the stepwise evolution.

**Figure 4:**
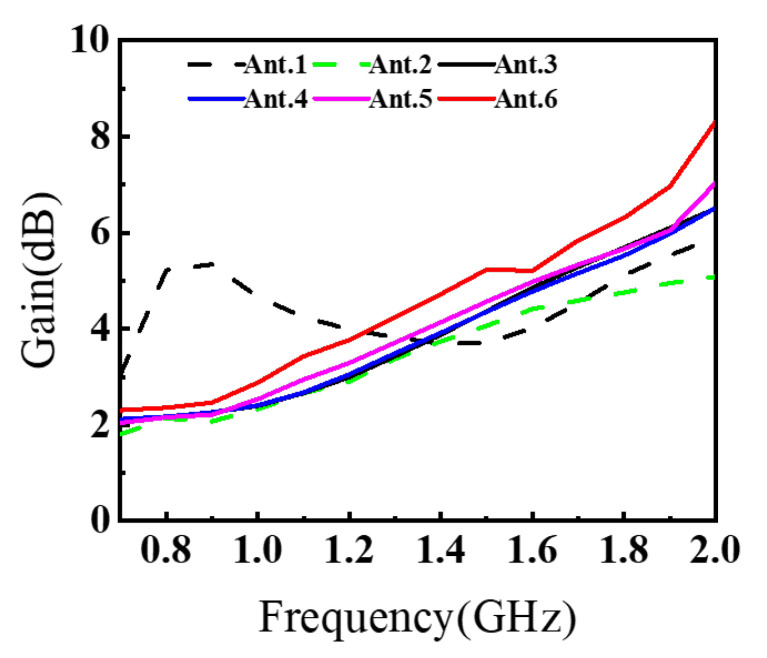
Simulated realized gain of Ant.1–Ant.6 during the stepwise evolution.

**Figure 5:**
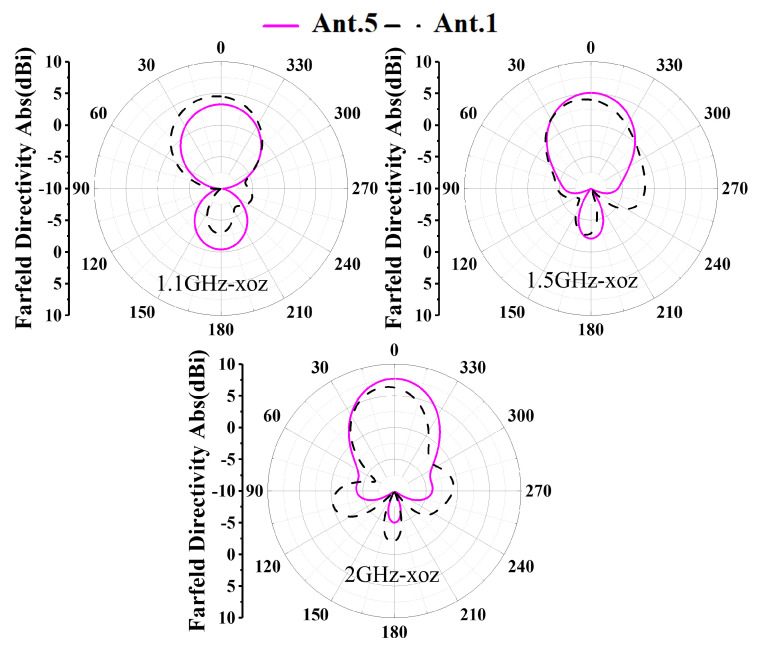
Gain simulated results of Ant.1 and Ant.5.

To clearly demonstrate the design process of the proposed antenna, Figure 2 illustrates six antenna configurations and their corresponding $S_{11}$. The AVA achieves miniaturization through these synergistic modifications. The conventional AVA (Ant.1) employs a standard AVA model, whose reflection coefficient $S_{11}$ performance is illustrated in Figure 3. While this design exhibits satisfactory operational characteristics above 1.2 GHz, it fails to meet impedance matching requirements below this frequency threshold, rendering it unsuitable for applications of GPR systems. In conventional AVA designs, the inclusion of feeder ports enhances impedance matching and bandwidth. However, for GPR systems, such a configuration introduces an undesirable radiation pattern deviation, adversely affecting target detection accuracy. To address this limitation, Step 1 implements a structural optimization by reducing the feed port through a symmetrical design approach. Specifically, the feed port of the AVA is reconfigured to achieve rotational symmetry about the antenna centerline. This modification not only lowers the minimum operating frequency but also corrects the radiation characteristics, ensuring improved alignment with GPR system requirements. In Ant.2, $S_{11}$ in the 1.1–1.4 GHz band exceed −10 dB. This is addressed in Step 2 by introducing interconnected elliptical structures and rectangular slots at the AVA’s upper section. The rectangular slot extends from the elliptical structure’s center to align with the antenna’s central axis, segmenting the copper layer on the right side of the ellipse. As Figure 3 indicates, Ant.3 significantly reduces $S_{11}$ below −10 dB across the 1.0–1.6 GHz band compared to Ant.2, while Figure 4 demonstrates notable gain improvement, particularly above 1.5 GHz. Ant.3 shows $S_{11}$ exceeding −10 dB beyond 1.8 GHz, which is resolved in Step 3 through slotting along the inner arc. This modification creates a meandering effect that lowers the fundamental resonant frequency and expands bandwidth. Since the inner arc serves as the primary current path, this adjustment significantly impacts antenna performance. As shown in Figure 3, a new resonance point emerges around 2 GHz, improving $S_{11}$ above 1.8 GHz to generally less than −10 dB while maintaining comparable gain characteristics. The simulated radiation pattern indicates elevated sidelobes before introducing the tilted aperture slots; this issue is mitigated in Step 4 by integrating four tilted rectangular slots at the flared aperture (Ant.5). This modification redirects current flow from the antenna edges, effectively enlarging the main lobe. Figure 5 confirms that Ant.5 achieves higher gain than Ant.1 between 1 and 2 GHz. Residual $S_{11}$ values above −10 dB near 1.8 GHz in Ant.5 are resolved in Step 5 through back-loaded parasitic patches. These patches enable capacitive coupling with adjacent copper layers, optimizing $S_{11}$ parameters to meet the less than −10 dB requirement across the 1.6–2 GHz band (Figure 3). The comprehensive optimization process culminates in Ant.6, which achieves balanced performance within a compact form factor through these synergistic modifications.

To quantitatively characterize the design evolution from Ant.1 to Ant.6, Table 1 reports a set of impedance-matching and radiation metrics under the standard −10 dB criterion. Specifically, $f_{L}$ and $f_{H}$ denote the lowest and highest frequencies that satisfy $S_{11}\leq -10$ dB for the longest continuous matched region, and thus $BW_{-10}^{\mathrm{cont}}$ denotes the bandwidth of the longest continuous matched interval, which measures the practically usable continuous operating band. In addition, $BW_{-10}^{\mathrm{total}}$ is the sum of all separated frequency intervals meeting $S_{11}\leq -10$ dB, while $n_{\mathrm{bands}}$ indicates how many such intervals exist (a smaller value implies a more continuous band). To explicitly quantify the residual mismatch around the critical region near 1.8 GHz, $\Delta f_{\mathrm{gap}@1.8}$ is defined as the width of the local gap where $S_{11}>-10$ dB. For radiation performance, G(1.8) and G(2.0) denote the realized gains at 1.8 GHz and 2.0 GHz, and ${\bar{G}}_{1-2}$ is the average realized gain over 1–2 GHz.

As summarized in Table 1, the intermediate designs (e.g., Ant.4 and Ant.5) still exhibit a split-band behavior, as evidenced by $n_{\mathrm{bands}}=2$ and a nonzero $\Delta f_{\mathrm{gap}@1.8}$, indicating a residual mismatch pocket around ∼1.8 GHz. After introducing the back-loaded parasitic patches (Ant.6), $\Delta f_{\mathrm{gap}@1.8}$ is reduced to zero and $n_{\mathrm{bands}}$ decreases to 1, which means that the previously separated −10 dB regions are merged into a single continuous matched band. Consequently, $BW_{-10}^{\mathrm{cont}}$ increases markedly, confirming that the final modification improves not only the depth of resonances but also the continuity of the usable bandwidth. Meanwhile, the gain metrics further improve in the upper portion of the band: both G(2.0) and ${\bar{G}}_{1–2}$ increase from Ant.5 to Ant.6, demonstrating that the final coupling optimization enhances radiation capability while maintaining compactness. To highlight the incremental contributions and quantify the claimed “synergy”, Table 2 presents the step-to-step improvements, where $\Delta BW_{-10}^{\mathrm{cont}}$, $\Delta f_{\mathrm{gap}@1.8}$, ΔG(2.0), and $\Delta {\bar{G}}_{1–2}$ explicitly show how the combined modifications eliminate the residual mismatch gap and simultaneously strengthen gain performance, providing a quantitative basis for the effectiveness of the overall design strategy.

Taken together, the Ant.4 → Ant.5 → Ant.6 progression quantitatively demonstrates the claimed synergy of the proposed co-design strategy: the residual mismatch pocket around ∼1.8 GHz is eliminated ($\Delta f_{\mathrm{gap}@1.8}$: 74 MHz → 41 MHz → 0), the continuous −10 dB bandwidth is extended ($BW_{-10}^{\mathrm{cont}}$: 1.05 GHz → 1.06 GHz → 1.26 GHz), and the upper-band gain is strengthened (G(2.0): 6.50 dBi → 6.99 dBi → 8.22 dBi; ${\bar{G}}_{1–2}$: 4.32 dBi → 4.53 dBi → 5.12 dBi). Meanwhile, the peak sidelobe level is reduced (e.g., in the XOZ cut at 2.0 GHz, PSLL improves from ∼−6.5 dB to ∼−12.3 dB), and the measured realized gain over 0.63–2.03 GHz exhibits a peak-to-peak variation of ΔG=5.4 dB, supporting the radiation stability claim beyond reflection coefficient improvement alone.

**Figure 6:**
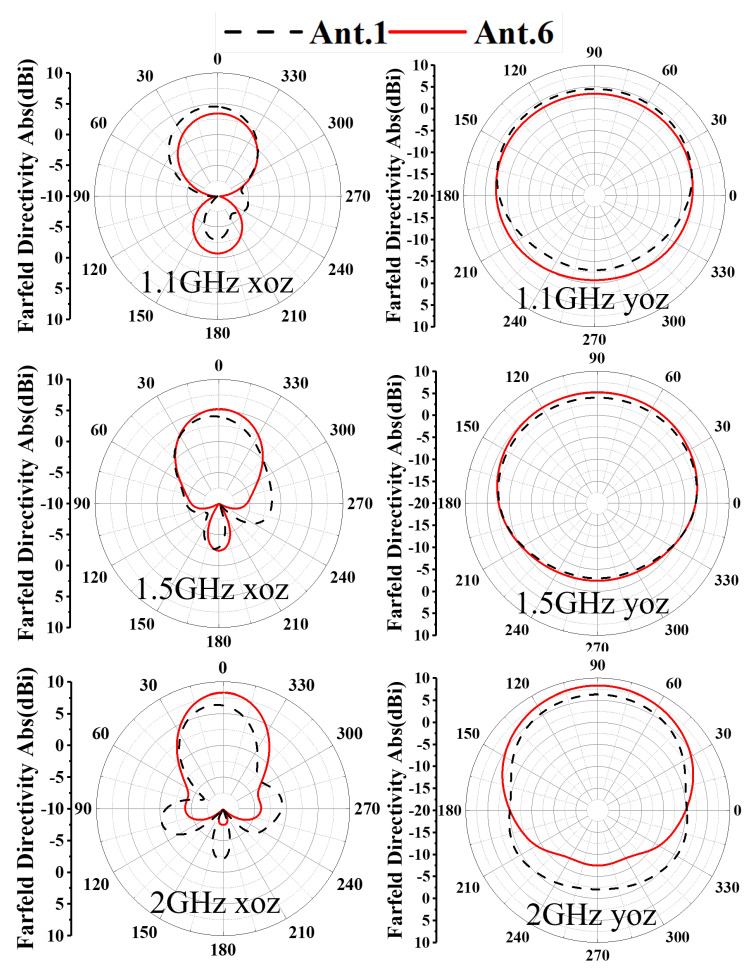
Simulated radiation pattern of Ant.1 and Ant.6.

To quantify the sidelobe/backlobe suppression, the peak sidelobe level (PSLL) is extracted from Figure 6 relative to the main-lobe peak. For the XOZ cut, the PSLL changes from approximately −6.2 dB (Ant.1) to −7.6 dB (Ant.6) at 1.5 GHz, and from about −6.5 dB (Ant.1) to −12.3 dB (Ant.6) at 2.0 GHz. For the YOZ cut, the PSLL changes from approximately −1.6 dB (Ant.1) to −2.8 dB (Ant.6) at 1.5 GHz, and from about −5.5 dB (Ant.1) to −8.3 dB (Ant.6) at 2.0 GHz. These results confirm a measurable sidelobe/backlobe reduction achieved by the overall step-by-step evolution (Ant.1–Ant.6), rather than being attributable to a single geometrical feature alone.

The implementation of aperture treatments on the upper and inner curved sections (primary surface current paths), when combined with a meandering mechanism, effectively reduces the antenna’s minimum operating frequency while broadening its bandwidth. The introduction of inclined rectangular slots at the horn aperture facilitates the expansion of the main-lobe width and concomitant suppression of sidelobe levels, thereby enhancing the overall gain characteristics. Furthermore, the incorporation of parasitic structures optimizes the antenna’s input impedance, ensuring stable impedance matching across operational bands and consequently improving gain performance. Collectively, these structural modifications not only elevate the operational efficiency and performance characteristics of the antenna but also broaden its range of potential application scope through enhanced frequency response and radiation pattern control.

### 2.3. Parameter Scanning Analysis

**Figure 7:**
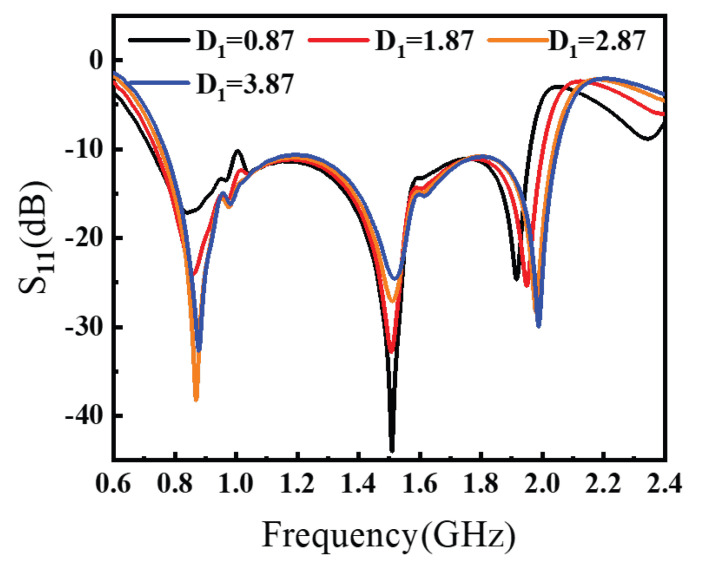
D1 scanning on $S_{11}$.

**Figure 8:**
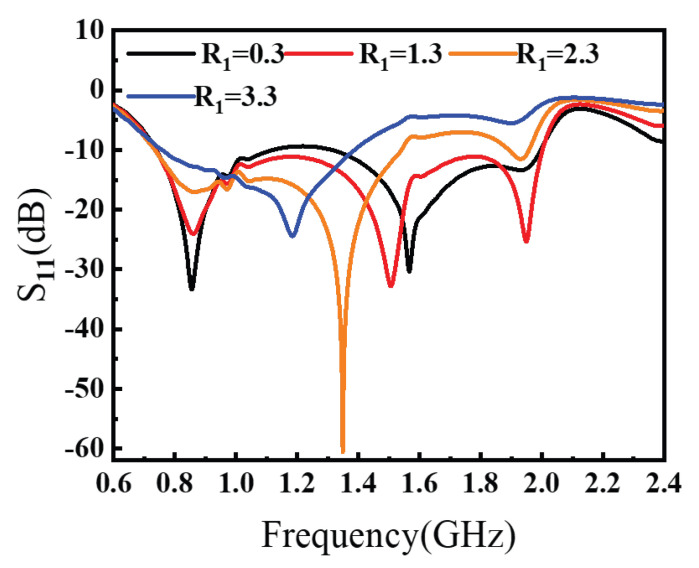
$R_{1}$ scanning on $S_{11}$.

In the optimization steps, the inclusion of two aperture-slot structures has a decisive impact on the impedance matching performance of the antenna. We study the slot dimension width $D_{1}$ and $R_{1}$ by analyzing the performance of $S_{11}$, and gain as shown in Figure 7 and Figure 8.

The critical parameter sweeps for antenna design are illustrated in Figure 7 and Figure 8. As shown in Figure 7, parameter $D_{1}$ significantly influences the S-parameters. Increasing $D_{1}$ shifts the antenna bandwidth toward higher frequencies. To maintain low-frequency operation, $D_{1}$ should be minimized. However, excessively small $D_{1}$ values degrade impedance matching near 1 GHz. Through comprehensive optimization, $D_{1}$ was ultimately set to 1.87 to balance bandwidth retention and matching performance. Figure 8 demonstrates the effect of $R_{1}$ on antenna characteristics. Enlarging $R_{1}$ improves impedance matching around 1.2 GHz but deteriorates it near 1.8 GHz. A trade-off between these two frequency bands is necessary. After iterative simulation and optimization, $R_{1}$ (referenced parameter) is finalized at 1.30 to achieve optimal dual-band performance.

### 2.4. Working Mechanism

**Figure 9:**
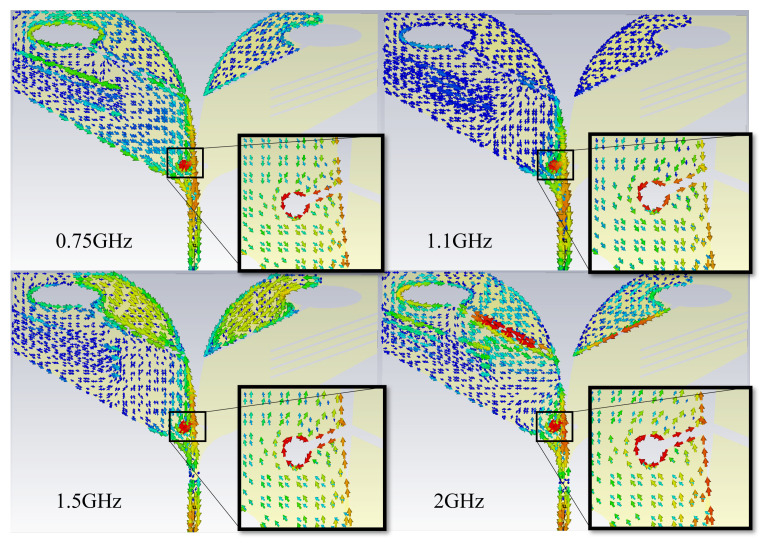
Surface current distributions at 0.75, 1.1, 1.5, and 2.0 GHz.

To thoroughly reveal the underlying electromagnetic working mechanisms responsible for the antenna’s miniaturization and broadband characteristics, the current distributions at representative operating frequencies (0.75 GHz, 1.1 GHz, 1.5 GHz, and 2.0 GHz) are analyzed in depth, as illustrated in Figure 9. The operating mechanism can be interpreted from full-wave simulated current distributions and field evolution. In general, the tapered AVA behaves as a traveling-wave radiator, where the electromagnetic energy propagates from the feed toward the aperture and radiates progressively as the impedance transforms to free space. In traditional designs, electromagnetic energy propagates along a smooth conductive path, radiating primarily at the aperture due to impedance transformation from the feed to free space. However, in the proposed antenna structure, strategically introducing slots and parasitic elements significantly alters the standard energy propagation mechanism, inducing multiple resonances and additional coupling effects.

## 3. Measured Results and Discussion

### Measured Results of Antenna

**Figure 10:**
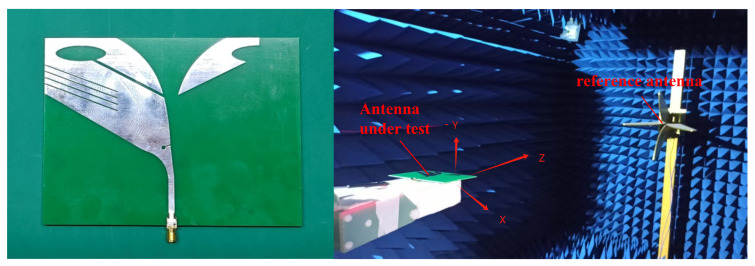
Prototype of the antenna. (**left**) Oblique view; (**right**) measurement environment.

**Figure 11:**
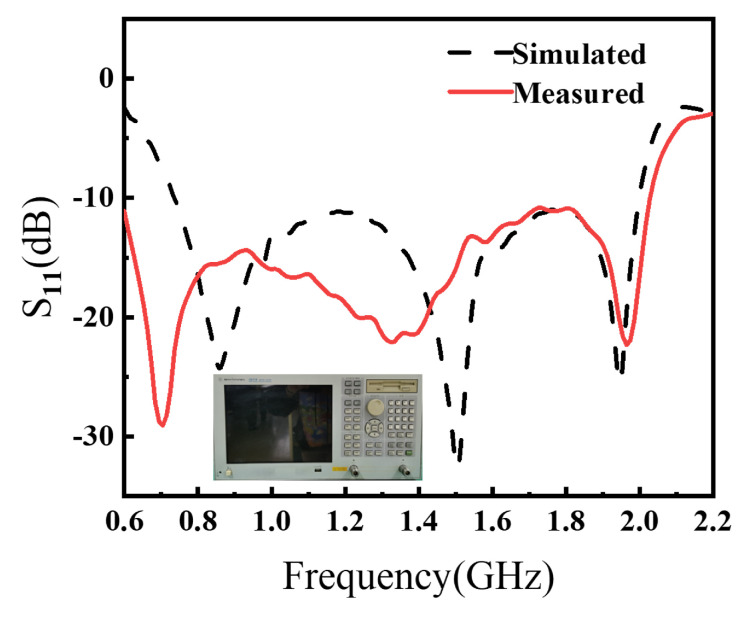
Simulated and measured results of $S_{11}$.

**Figure 12:**
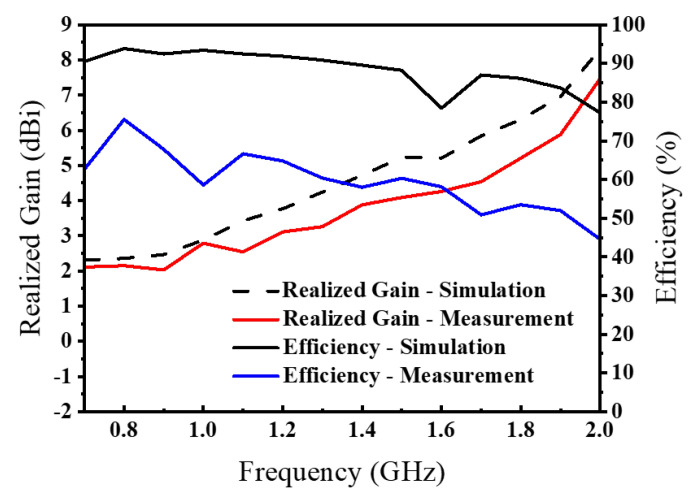
Simulated and measured realized gain and radiation efficiency.

**Figure 13:**
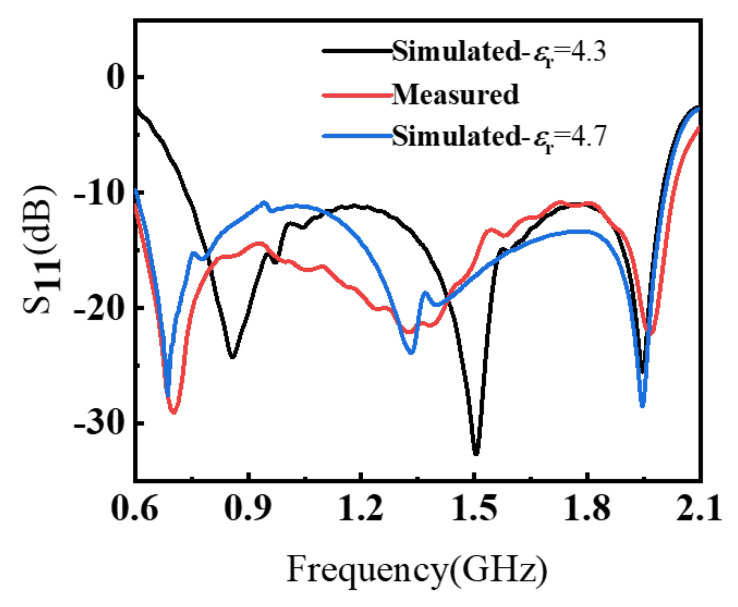
Simulated and measured reflection coefficient of the proposed antenna.

Figure 10(left) depicts the fabricated antenna prototype. As shown in Figure 10(right), the impedance and far-field radiation characteristics were measured in an anechoic chamber using a vector network analyzer (VNA, Agilent/Keysight E5071B, Keysight, Technologies, Santa Rosa, CA, USA). The simulated and measured results are presented in Figure 11 and Figure 12. The measured $S_{11}$ remains below −10 dB from 0.63 to 2.03 GHz, corresponding to a relative impedance bandwidth of 105.26%. Compared with the simulation, the measured impedance bandwidth is slightly wider, while the measured realized gain is slightly lower, which is mainly attributed to practical dielectric/conductor losses and measurement/assembly tolerances. To further clarify the remaining simulation–measurement mismatch, Figure 13 highlights the discrepancy in the 0.6–1.0 GHz band, where the measurement exhibits a deeper resonance near 0.7 GHz but a different response shape. The detailed design are provided in the Supplementary Materials.

From an engineering perspective, the low-frequency input impedance is particularly sensitive to the effective coax-to-microstrip launch and its parasitics. Although the full-wave model includes the SMA connector and the coax-to-microstrip transition (with the port reference plane defined at the connector input), small differences between the idealized model and the practical assembly can still introduce residual series inductance and shunt capacitance. Typical contributors include the solder-joint geometry and fillet size, connector manufacturing tolerance, imperfect ground return around the launch region, and the proximity/coupling of the fixture and coax cable during measurement. These effects can reshape the resonance and slope of $S_{11}$ in the electrically small regime, explaining the observed response differences around 0.7 GHz.

In addition, commercial FR-4 exhibits batch- and frequency-dependent variability in both $\epsilon_{r}$ and loss tangent tanδ. A slight deviation in the effective $\epsilon_{r}$ changes the electrical length of the current path and shifts the low-frequency resonance, while an increased effective tanδ and copper/transition losses reduce radiation efficiency and thus lower the measured gain relative to simulation. In our parametric check, an effective $\epsilon_{r}$ close to 4.7 provides the closest agreement with the measured $S_{11}$ trend for the fabricated prototype. Finally, geometric tolerances on the narrow slot/arc features near the feed and inner current path (e.g., the key slot widths and arc radii) can cause noticeable impedance perturbations; this is consistent with the fact that the mismatch is concentrated in the low-frequency portion where the antenna is most sensitive to small variations in current-path length and launch parasitics. Overall, the discrepancy is mainly attributed to residual connector/launch parasitics under practical measurement conditions, together with FR-4 material variability and fabrication/assembly tolerances.

**Figure 14:**
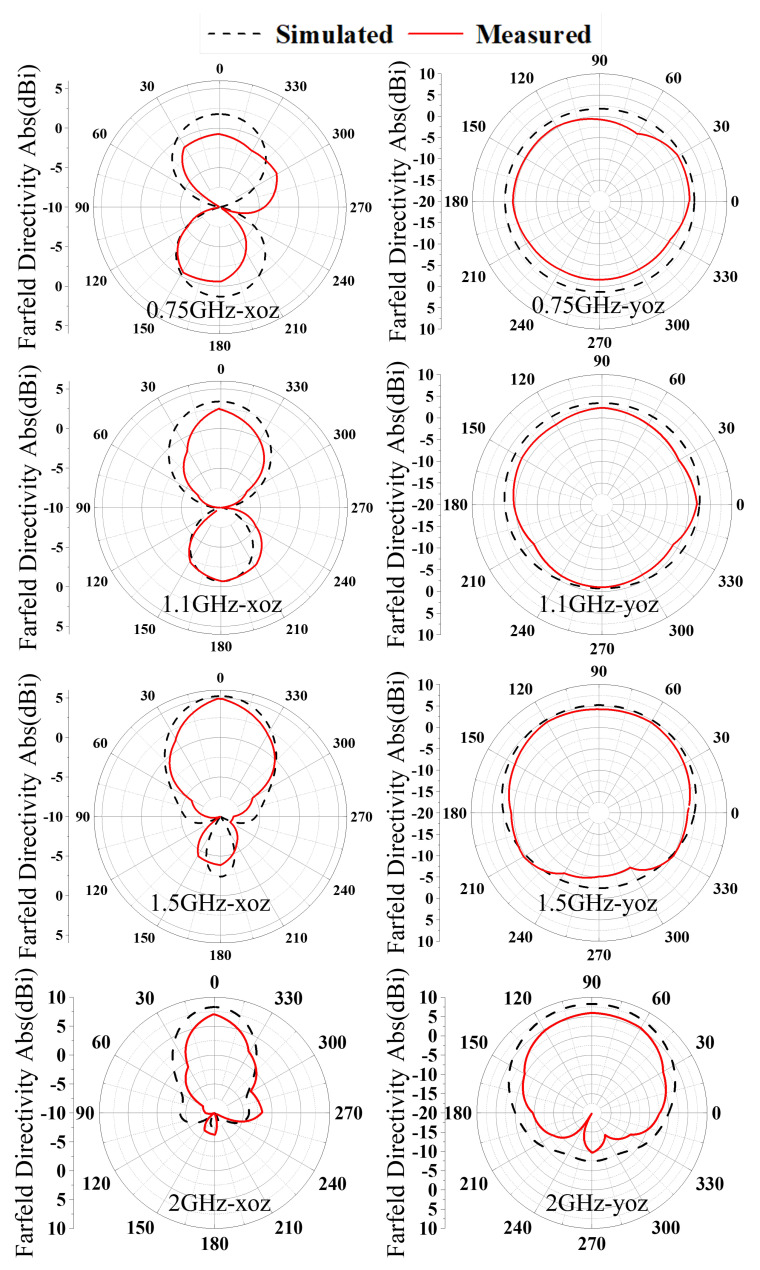
Simulated and measured far-field radiation patterns.

As illustrated in Figure 14, the gain radiation patterns of the AS-AVA in the *xoz*- and *yoz*-planes exhibit negligible deviations between experimental and simulated results. Additional analyses of gain characteristics at other frequencies demonstrate consistent stability across the entire operational bandwidth. The antenna exhibits relatively lower gain in the low-frequency range with gradual enhancement as frequency increases, a phenomenon attributable to the combined effects of insertion loss in the feeding network, current-path alterations induced by slotting and perforation processes, and the gain superposition of coupled patches at mid-to-high frequencies. This observed frequency-dependent gain variation conforms to theoretical expectations and remains within acceptable operational limits.

**Figure 15:**
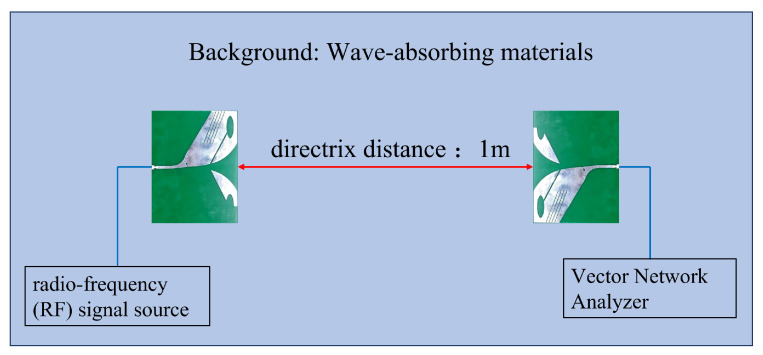
Measurement setup for group delay.

**Figure 16:**
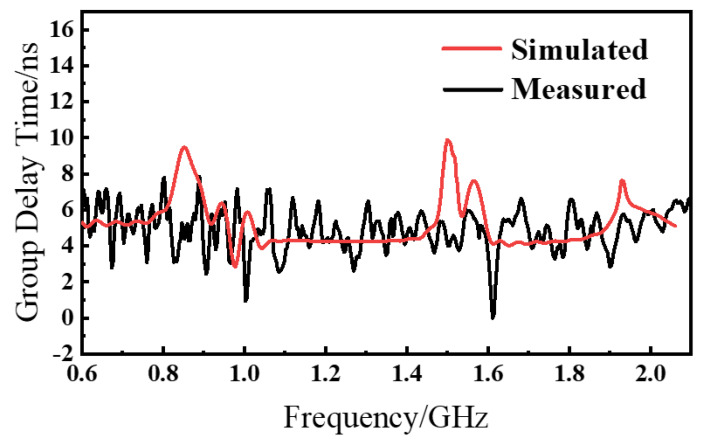
Simulated and measured group delay.

The AS-AVA is designed for transmitting and receiving stepped-frequency continuous-wave (SFCW) signals; therefore, a stable group delay is required to preserve time-domain fidelity. The group delay was measured using the setup in Figure 15, where the antenna spacing was set to 1 m and the excitation covered 0.6–2.1 GHz. A vector network analyzer (VNA) was used to obtain the group delay response. As shown in Figure 16, the measured group delay remains relatively stable within the operating band, ranging from 4 to 8 ns, which indicates good time-domain fidelity and supports stable radar imaging.

Table 3 presents a performance comparison between the proposed AS-AVA and representative antipodal Vivaldi-type designs. The results indicate that the AS-AVA achieves an ultra-wide measured impedance bandwidth of 105.26% (0.63–2.03 GHz) while maintaining a compact electrical size of 0.33$\lambda_{0}\;\times$ 0.23$\lambda_{0}$ (referenced to the lowest operating frequency). This combination is particularly advantageous for GPR applications, where low-frequency coverage supports penetration depth and compact integration is required for portable or drone-mounted platforms. Although the realized gain at the lower end is limited due to the electrically small aperture and practical losses, the measured gain increases with frequency and reaches about 7.5 dBi near the upper end of the band, which is sufficient for typical GPR sensing scenarios. Over the measured operational band (0.63–2.03 GHz), the realized gain varies from 2.1 to 7.5 dBi, corresponding to a peak-to-peak gain variation of ΔG=5.4 dB, which provides a quantitative metric for the claimed gain stability. Overall, the comparison confirms that the proposed antenna provides a competitive trade-off between bandwidth, size, and radiation performance for compact sub 3 GHz GPR systems.

The enhanced performance of the AS-AVA is enabled by the proposed structural modifications, which achieve a favorable balance between wide impedance bandwidth and compact size. Compared with representative designs in the literature, the proposed antenna provides improved low-frequency coverage within a reduced footprint, which is beneficial for portable or space-constrained GPR platforms. The operating band of 0.63–2.03 GHz supports practical detection under different subsurface conditions and target depths, while the compact form factor facilitates integration into handheld or drone-mounted GPR systems.

## 4. Antenna Applications

### 4.1. GPR Parameters

In the validation experiments, the proposed antenna pair was integrated into an SFCW GPR testbed. The radar transmits a set of discrete single-tone frequencies and coherently records the frequency-domain response. The time-domain A-scan is obtained by applying an inverse discrete Fourier transform (IDFT), implemented via an inverse fast Fourier transform (IFFT), to the sampled frequency-domain data. Sequential A-scans acquired along the scan track are stacked to form a B-scan image.

For reproducibility, Table 4 summarizes the key radar host and measurement settings, including the SFCW waveform configuration (start/stop frequencies, number of tones, frequency step, and coherent receive window per tone), receiver/acquisition description, and the main scanning settings (trace spacing, Tx–Rx spacing, and scan mode). Specifically, the waveform uses $f_{\mathrm{start}}=0.7$ GHz, N=100, and Δf=10 MHz (thus $f_{\mathrm{stop}}=f_{\mathrm{start}}+\left(N-1\right)\Delta f=1.69$ GHz), with a coherent receive window per tone of $T_{0}=10^{-7}$ s. The synthesized bandwidth is B=(N−1)Δf=0.99 GHz. The receiver is implemented using a self-developed ADC-based acquisition module, and the scan is performed with an approximate spatial step of Δx≈1 mm per trace. The experiments were conducted on a laboratory SFCW-GPR platform (commercial signal generation combined with a self-built coherent receiver), rather than a fully commercial radar host.

### 4.2. Data Processing Algorithm

To enable readers to evaluate the intrinsic raw-data quality, we present the raw B-scan (directly after IFFT-based range conversion) alongside the processed B-scan. In the raw B-scan, the direct coupling wave and the ground/interface reflection can be observed in the early-time region, while buried targets generate hyperbolic signatures. The processing pipeline is summarized as follows:

1. Time-zero alignment and DC removal. A time-zero correction is applied to align the direct-wave arrival, and a DC component is removed:
  (2) $$A_{0}\left(x,t\right)=A\;\left(x,t+t_{z}\right)-\frac{1}{T}\int_{0}^{T}A\;\left(x,\tau \right)\;d\tau ,$$
  where $t_{z}$ denotes the time-zero shift and *T* is the record length.
2. Background removal (clutter suppression). A background trace is estimated by averaging all traces and is subtracted from each trace:
  (3) $$B\left(x,t\right)=A_{0}\left(x,t\right)-\frac{1}{M}\sum_{m=1}^{M}A_{0}\left(x_{m},t\right),$$
  where $A_{0}\left(x,t\right)$ is the time-aligned B-scan and *M* is the number of traces.
3. Band-pass filtering. Out-of-band noise is suppressed by applying a band-pass filter along the fast-time axis:
  (4) $$B_{f}\left(x,t\right)=\left(B\left(x,\cdot \right)*h_{\mathrm{bp}}\left(\cdot \right)\right)\left(t\right),$$
  where $h_{\mathrm{bp}}\left(t\right)$ is the impulse response of the band-pass filter and ∗ denotes convolution.
4. Time-varying gain compensation. To counteract attenuation, a depth/time-dependent gain is applied:
  (5) $$A_{g}\left(x,t\right)=g\left(t\right)\;B_{f}\left(x,t\right),$$
  where g(t) is a monotonic gain function of time (depth).
5. Visualization. The processed B-scan is displayed in pseudo-color using a consistent dynamic range via clipping and normalization:
  (6) $$I\left(x,t\right)=\frac{\mathrm{clip}\;\left(A_{g}\left(x,t\right),\;a_{\min},\;a_{\max}\right)-a_{\min}}{a_{\max}-a_{\min}},$$
  where clip(·) truncates values to $\left[a_{\min},a_{\max}\right]$ and I(x,t)∈[0,1] is mapped to a pseudo-color colormap; key features (direct coupling, surface/interface reflection, and target hyperbolas) are annotated.

### 4.3. GPR Experiment

**Figure 17:**
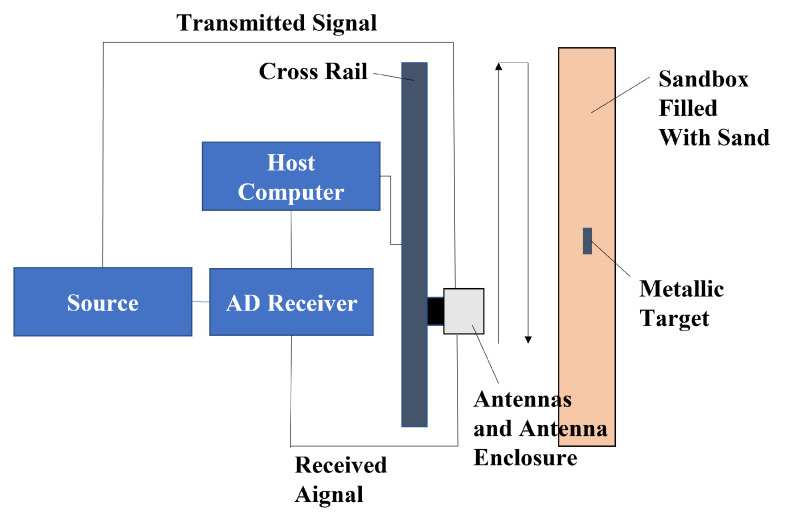
Schematic diagram of the experimental site.

**Figure 18:**
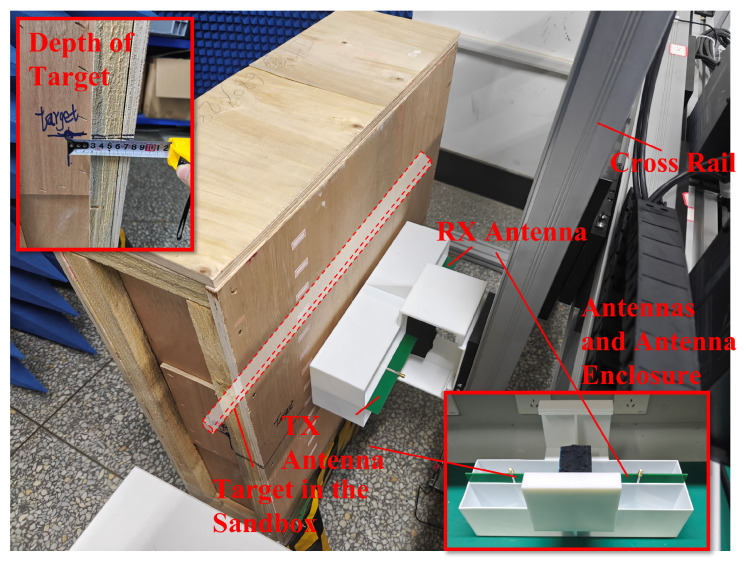
Experimental site.

**Figure 19:**
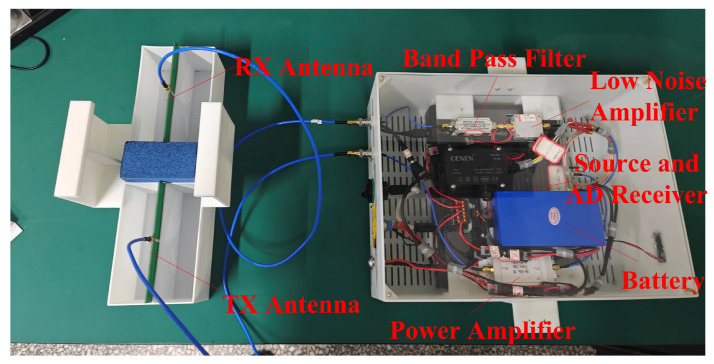
Signal chain for the GPR measurement.

To further validate the performance of AS-AVA, an experiment was designed to map metal bars embedded in sandy soil at a burial depth of 7.69 cm. As shown in Figure 17 and Figure 18, the experimental setup consists of two AS-AVAs fixed on a guide rail through antenna housings. As shown in Figure 19, the antenna is connected to this signal link for testing. The Tx (transmitting) antenna was connected to a signal generator, while the Rx (receiving) antenna was linked to the radar’s receiver. Due to the limitations of laboratory equipment, the signal used for the experiment was a step-frequency continuous-wave signal from 0.7 to 1.69 GHz. To verify the antennas’ operability, the antennas and their housings were driven by the sliding rail to perform a constant-speed reciprocal motion (one complete up-and-down cycle). Throughout this movement, radar signals were continuously transmitted and received. During measurement, each complete cycle of signal transmission and reception constitutes a discrete measurement. The one-dimensional time-domain waveform obtained from a single measurement is defined as an A-scan, which encapsulates the electromagnetic reflection characteristics of subsurface media at a specific spatial position. By systematically accumulating sequential A-scans along the detection trajectory, these individual measurements undergo spatiotemporal alignment to form a two-dimensional cross-sectional representation, designated as a B-scan image. After signal processing, the measured B-scan image was obtained, and the experimental results are illustrated in the figure.

**Figure 20:**
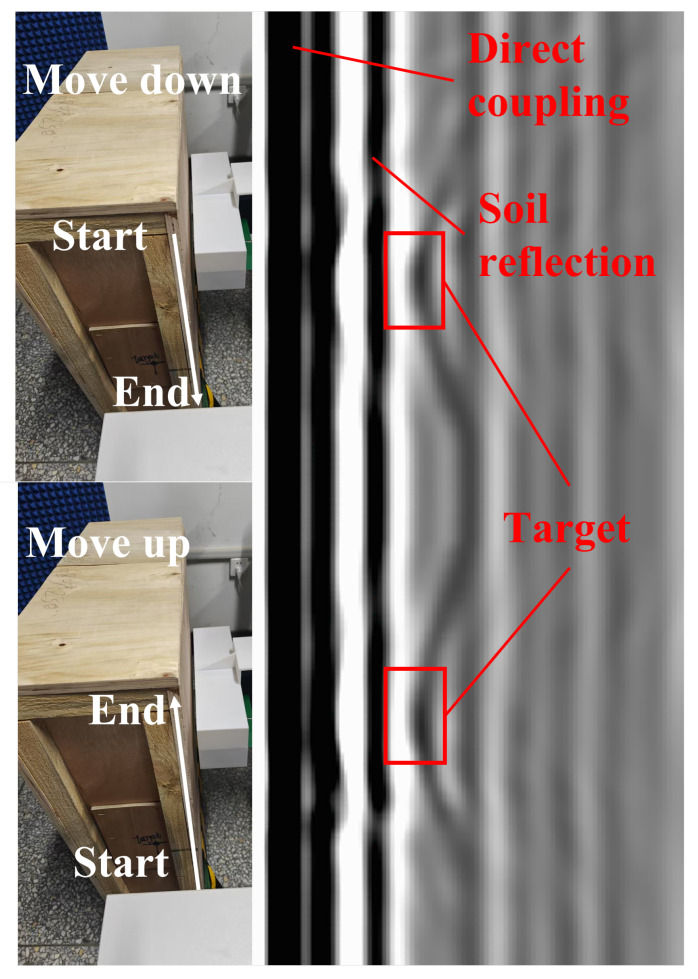
Experimental procedure and the raw B-scan image measurement.

**Figure 21:**
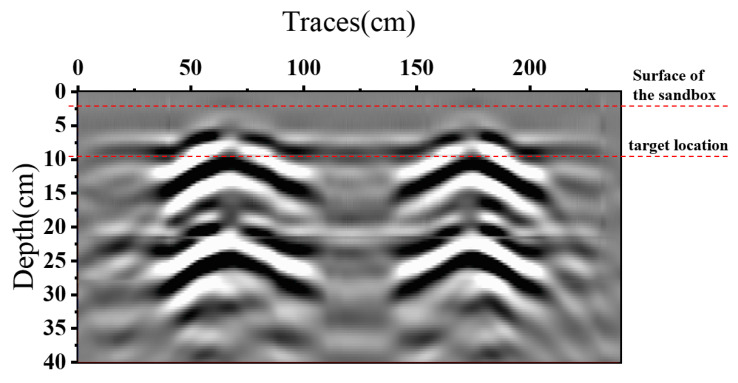
Processed B-scan after time-zero/DC removal, background subtraction, filtering, and gain compensation.

Figure 20 shows the raw B-scan image, while Figure 21 presents the processed B-scan after signal processing. Owing to the uniform motion of the antenna along the scan line, subsurface targets exhibit the characteristic hyperbolic signatures in the B-scan. In the raw data (Figure 20), strong early-time components are dominated by the direct Tx-Rx coupling and near-surface/interface reflections, which partially mask weaker target responses. After applying the adopted signal processing steps to suppress the coupling wave and background components, the upper horizontal feature in Figure 21 mainly corresponds to the near-surface/interface reflection from the sandbox container, and the two clear hyperbolas are associated with the experimental targets. As shown in Figure 21, the apex of each hyperbola indicates the target depth, which is estimated to be 7.51 cm with an error of 0.18 cm compared with the ground-truth measurement. These results validate the effectiveness of the proposed antenna in GPR systems and demonstrate its capability to resolve subsurface features with quantifiable accuracy. The obtained depth error (1.8 mm) is considered reasonable for a controlled sandbox scenario with a short propagation path, a strong target signature, and a fixed Tx–Rx geometry, provided that the effective wave velocity is approximately known. The remaining discrepancy is mainly attributed to (i) uncertainty in the effective propagation velocity of the sand/backfill (including spatial non-uniformity and moisture dependence), (ii) finite temporal resolution and peak-picking ambiguity after IFFT-based range conversion and post-processing, and (iii) small systematic offsets related to time-zero alignment and the reference definition of antenna height and Tx–Rx spacing.

**Figure 22:**
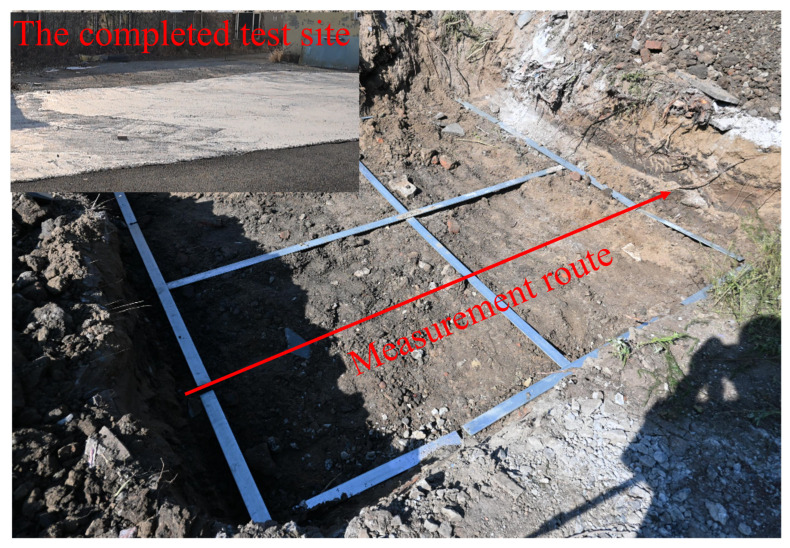
Grid-shaped iron-bar target layout used for controlled validation, where metallic bars are arranged in a predefined grid within the prepared test pit.

**Figure 23:**
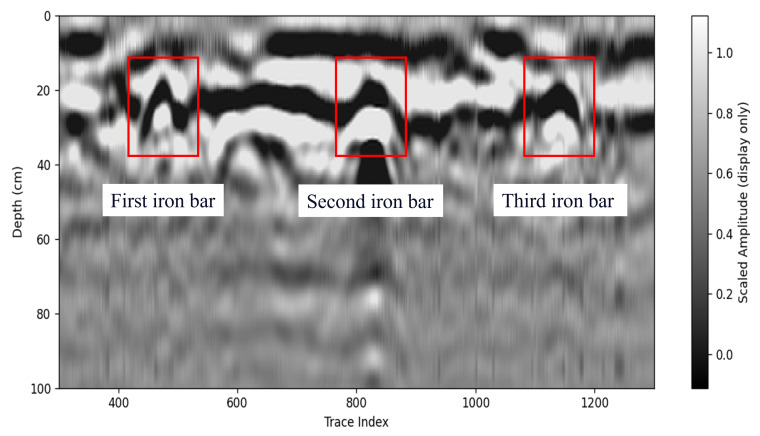
Processed B-scan result for the grid-shaped iron-bar experiment after time-zero/DC removal, background subtraction, filtering, and gain compensation, where the iron bars produce clear hyperbolic signatures.

Figure 22 and Figure 23 present a controlled experiment for detecting grid-shaped iron bars. As shown in Figure 22, the survey area was prepared as a rectangular test pit, and the metallic targets were arranged in a grid within the designated region. During the measurement, the antenna pair was moved along the predefined scan direction with a fixed spatial step, and consecutive A-scans were stacked to form the B-scan image. Figure 23 shows the corresponding B-scan result after signal processing for visualization. In the B-scan, strong early-time responses are mainly associated with direct coupling and near-surface reflections, while the embedded iron bars produce clear hyperbolic signatures along the scan axis. These localized high-amplitude responses and their characteristic hyperbolic patterns indicate that the proposed antenna-enabled GPR setup can reliably capture the electromagnetic signatures of shallow metallic targets under the controlled grid arrangement.

**Figure 24:**
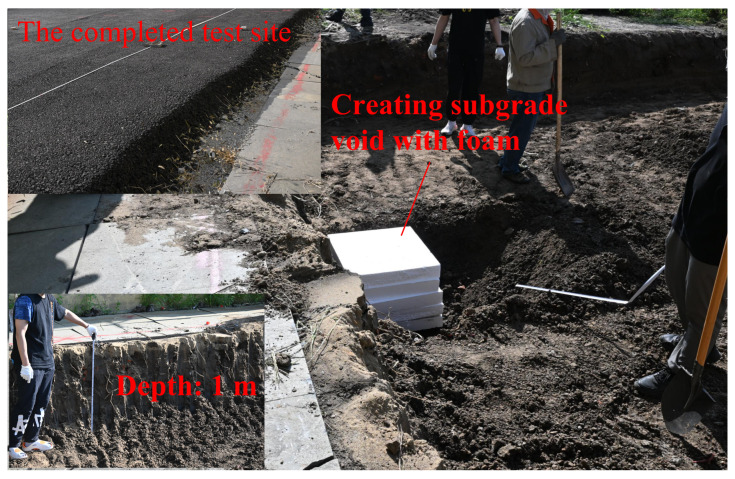
Subgrade-void test scene (approximate depth ∼1 m), where a void-like cavity is created using foam blocks to introduce a strong impedance contrast relative to the surrounding soil.

**Figure 25:**
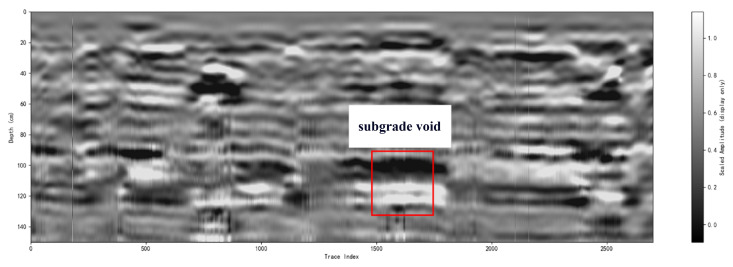
Processed B-scan result for the subgrade-void experiment; the void region (red box) appears as a coherent high-contrast anomaly that disrupts the background reflections.

Figure 24 and Figure 25demonstrate a subgrade-void validation experiment. As illustrated in Figure 24, a representative subgrade void was artificially created by placing foam blocks inside an excavated cavity, with an approximate depth of 1 m. The survey was then conducted along the ground surface above the void region using the same scanning strategy. Figure 25 depicts the processed B-scan result, where the void region (marked by the red box) exhibits a distinct localized response compared with the surrounding background. Specifically, the void-related signature appears as a coherent, high-contrast anomaly that disrupts the continuity of the background reflections, which is consistent with the strong impedance contrast introduced by a foam-filled cavity. The clear visibility of this anomaly confirms the practical capability of the proposed antenna for subsurface defect identification in realistic subgrade-like scenarios. Regarding band selection, the adopted sub 3 GHz range represents a practical penetration–resolution compromise for near-surface sensing at depths on the order of 1 m. The lower-frequency portion contributes more to penetration in lossy soils, while the higher-frequency portion improves range/feature resolution and enhances the visibility of localized anomalies. Therefore, combining these components in the stepped-frequency synthesis helps maintain detectability of the void signature while preserving adequate imaging sharpness.

## 5. Conclusions

This paper presents a compact broadband aperture-slot antipodal Vivaldi antenna (AS-AVA) for sub 3 GHz GPR systems. Unlike the extensive AVA literature at higher frequencies, compact AVAs below 3 GHz—especially for GPR—are relatively scarce because the longer wavelength makes miniaturization difficult without degrading radiation performance. To address this gap, the proposed design is developed under a radiation stability-driven co-design strategy that jointly considers low-frequency extension, impedance continuity, and robust radiation behavior.

The antenna uses aperture treatments with controlled meandering to extend the low-frequency edge, tilted aperture slots to suppress excessive edge currents and reduce sidelobes for more stable gain/patterns, and back-loaded parasitic patches for coupling-based impedance refinement. The fabricated prototype achieves $S_{11}<-10$ dB from 0.63 to 2.03 GHz with a compact electrical size of $0.33\lambda_{0}\times 0.23\lambda_{0}\times 0.004\lambda_{0}$ ($\lambda_{0}$ at the lowest frequency) and maintains stable radiation characteristics. Sandbox and outdoor GPR experiments further confirm clear target signatures, demonstrating suitability for portable and drone-mounted GPR applications such as underground utility mapping and infrastructure inspection.

## Figures and Tables

**Table 1:** Quantitative comparison of Ant.1–Ant.6 during the design evolution.

Ant.	Key Modification	$f_{L}$	$f_{H}$	${\mathrm{BW}}_{-10}^{\mathrm{cont}}$	${\mathrm{BW}}_{-10}^{\mathrm{total}}$	$n_{\mathrm{bands}}$	$\Delta f_{\mathrm{gap}@1.8}$	G(1.8)	G(2.0)	${\bar{G}}_{1–2}$
		(GHz)	(GHz)	(GHz)	(GHz)		(MHz)	(dBi)	(dBi)	(dBi)
1	Baseline AVA	0.74	2.40	0.78	1.26	3	0	4.88	5.82	4.63
2	Symmetric reduced feed	0.70	2.40	1.00	1.35	2	0	4.74	5.07	3.89
3	Upper ellipse + rectangular slot	0.71	2.40	0.55	1.30	5	0	5.54	6.50	4.32
4	Inner-arc meander slot	0.76	2.07	1.05	1.24	2	74	5.51	6.50	4.32
5	Tilted aperture slots (4×)	0.75	2.06	1.06	1.27	2	41	5.65	6.99	4.53
6	+Back-loaded parasitic patches	0.74	2.00	1.26	1.26	1	0	6.31	8.22	5.12

**Table 2:** Incremental improvements to quantify the synergy (Ant.4 → Ant.5 → Ant.6).

Transition	$\Delta {\mathrm{BW}}_{-10}^{\mathrm{cont}}$ (GHz)	$\Delta f_{\mathrm{gap}@1.8}$ (MHz)	ΔG(2.0) (dB)	$\Delta {\bar{G}}_{1–2}$ (dB)
Ant.4 → Ant.5	+0.011	74 → 41	+0.49	+0.21
Ant.5 → Ant.6	+0.208	41 → 0	+1.24	+0.59

**Table 3:** Performances of the proposed antenna and referenced antennas.

Ref.	Bandwidth (GHz)	Electric Size ($\lambda_{0}$)	Gain/Lowest Point (dBi)	Gain/Highest GHz (dBi)
[23]	1.5–3	0.75 × 0.75	6.6	7.5
[24]	0.47–2.8	0.71 × 1.06	3	7
[25]	0.3–2	0.45 × 0.6	4.4	11.5
This work	0.63–2.03	0.33 × 0.23	2.	7.5

**Table 4:** Radar host and measurement parameters used in the SFCW-GPR experiments.

Parameter	Value/Setting
Waveform type	Stepped-frequency continuous wave (SFCW)
Start frequency $f_{\mathrm{start}}$	0.7 GHz
Number of tones *N*	100
Frequency step Δf	10 MHz
Stop frequency $f_{\mathrm{stop}}$	1.69 GHz
Synthesized bandwidth *B*	0.99 GHz
Coherent receive window per tone $T_{0}$	$10^{-7}$ s
Receiver type	Self-developed coherent receiver + ADC acquisition module
Trace spacing (spatial step) Δx	∼1 mm per trace
Antenna configuration	Two identical antennas (Tx/Rx)
Tx–Rx spacing (B-scan experiment)	206.82 mm
Scan mode	Constant-step linear scan; A-scans stacked to form B-scan
Processing summary	Time-zero/DC removal + background removal +
	filtering + gain compensation + normalization

## Data Availability

The original contributions presented in this study are included in the article/Supplementary Material. Further inquiries can be directed to the corresponding author.

## Supplementary Materials

The following supporting information can be downloaded at: https://www.mdpi.com/article/10.3390/s26030810/s1, Figure S1. SMA modeling. Figure S2. SMA modeling. Figure S3. Assembly of the SMA connector and the antenna (without soldering). Figure S4. Assembly of the SMA connector and the antenna (without soldering). Figure S5. Antenna modeling after soldering. Figure S6. Antenna modeling after soldering.

## Abbreviations

The following abbreviations are used in this manuscript:

GPR	ground-penetrating radar
UWB	ultra-wideband
AVA	antipodal Vivaldi antenna
SFCW	stepped-frequency continuous wave
AS-AVA	aperture-slot antipodal Vivaldi antenna
