# Supplementary material for: A Compact Aperture-Slot Antipodal Vivaldi Antenna for GPR Systems
Source: Sensors (Basel). 2026 Jan 26;26(3):810. doi: 10.3390/s26030810 (PMC12899646; doi:10.3390/s26030810)
Supplement: Supplementary file 1 [file sensors-26-00810-s001.zip › sensors-4047411-supplementary.pdf]

Supplementary Materials: A Compact Aperture-Slot Antipodal Vivaldi Antenna for GPR Systems

Feng Shen [1], Ninghe Yang [2] , Chao Xia [1,*] ,Tong Wan[1] and Jiaheng Kang [1]

1. Antenna simulation model with an SMA connector

1.1. Simulation of the SMA connector

In the experiment, we used a standard SMA-KE connector with external thread and internal pin that we purchased, which theoretically should sufficiently meet the requirements of the designed antenna. The modeling is shown in the Fig.S1 and Fig.S2.The yellow parts are copper, with a relative permittivity of 1; the white parts are plastic, with a relative permittivity of 2.1.

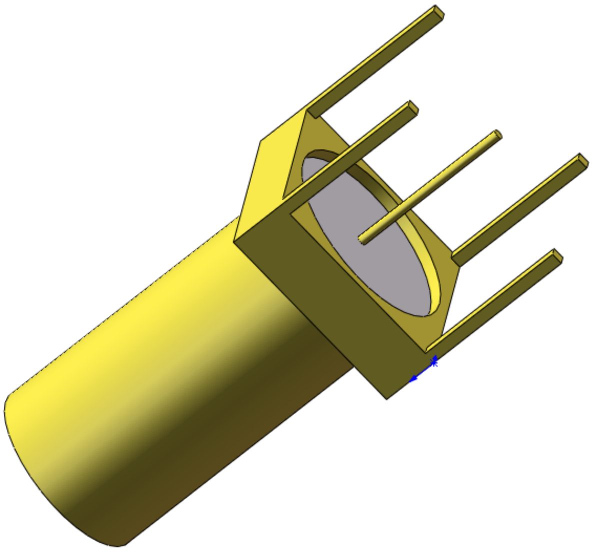

Figure S1. SMA modeling

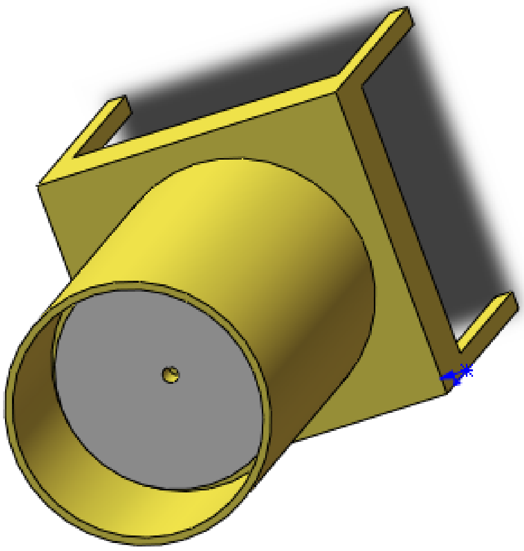

Figure S2. SMA modeling

1.2. Connection between the antenna and the SMA connector

The specific installation method is shown in Fig.S3 and Fig.S4, the inner pin of the SMA connector is soldered to the feed port on one side of the antenna, while the outer shell (ground) is soldered to the feed port on the opposite side of the antenna.

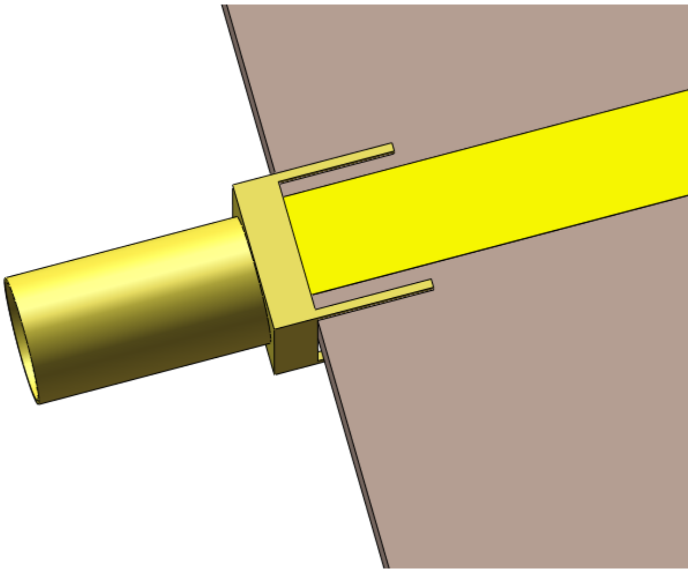

Figure S3. Assembly of the SMA connector and the antenna (without soldering)

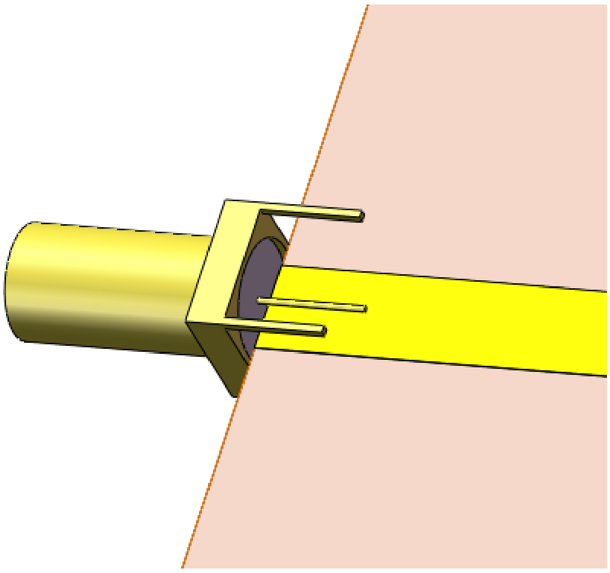

Figure S4. Assembly of the SMA connector and the antenna (without soldering)

1.3. Antenna feeding method

The post-soldering model is shown in Fig.S5 and Fig.S6, where the SMA connector is connected to the antenna via solder joints. The SMA connector geometry and the coax-to-microstrip transition are included; the antenna is excited through a coaxial port at the SMA input reference plane.

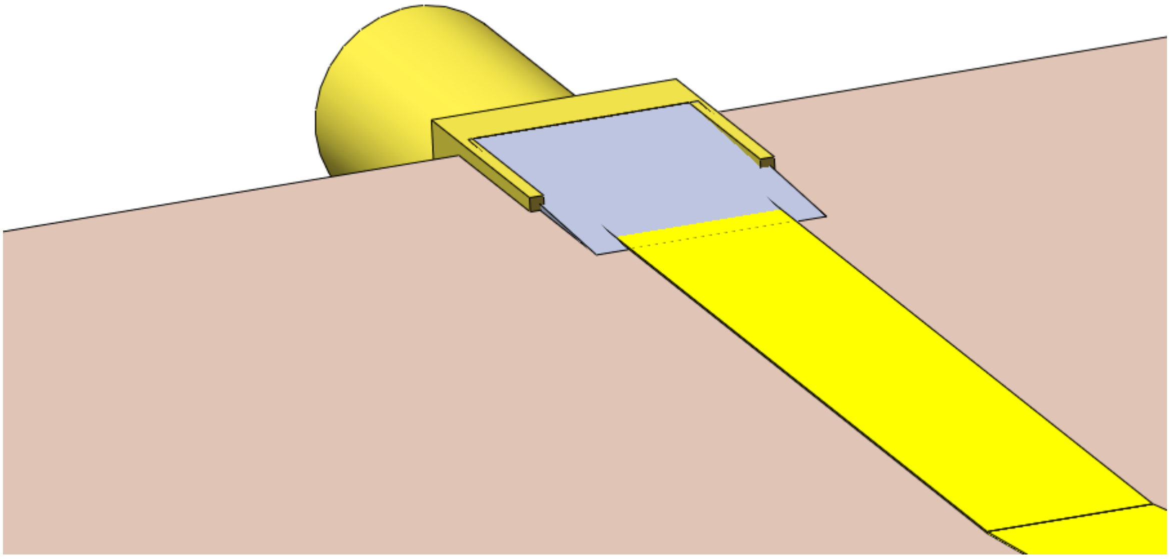

Figure S5. Antenna modeling after soldering

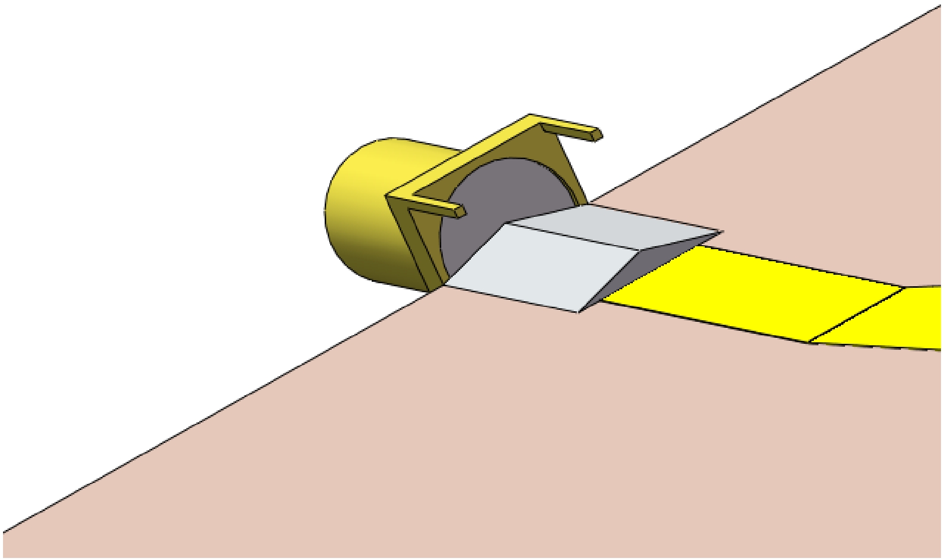

Figure S6. Antenna modeling after soldering

Disclaimer/Publisher's Note: The statements, opinions and data contained in all publications are solely those of the individual author(s) and contributor(s) and not of MDPI and/or the editor(s). MDPI and/or the editor(s) disclaim responsibility for any injury to people or property resulting from any ideas, methods, instructions or products referred to in the content.
